# In-depth performance analysis of an EEG based neonatal seizure detection algorithm

**DOI:** 10.1016/j.clinph.2016.01.026

**Published:** 2016-05

**Authors:** S. Mathieson, J. Rennie, V. Livingstone, A. Temko, E. Low, R.M. Pressler, G.B. Boylan

**Affiliations:** a Academic Research Department of Neonatology, Institute for Women’s Health, University College London, London, United Kingdom; b Neonatal Brain Research Group, Irish Centre for Fetal and Neonatal Translational Research, Department of Paediatrics and Child Health, University College Cork, Ireland; c Department of Electrical and Electronic Engineering, University College Cork, Ireland; d Department of Clinical Neurophysiology, Great Ormond Street Hospital, London, United Kingdom

**Keywords:** Automated seizure detection, Neonatal seizures

## Abstract

**Objective:**

To describe a novel neurophysiology based performance analysis of automated seizure detection algorithms for neonatal EEG to characterize features of detected and non-detected seizures and causes of false detections to identify areas for algorithmic improvement.

**Methods:**

EEGs of 20 term neonates were recorded (10 seizure, 10 non-seizure). Seizures were annotated by an expert and characterized using a novel set of 10 criteria.

ANSeR seizure detection algorithm (SDA) seizure annotations were compared to the expert to derive detected and non-detected seizures at three SDA sensitivity thresholds. Differences in seizure characteristics between groups were compared using univariate and multivariate analysis. False detections were characterized.

**Results:**

The expert detected 421 seizures. The SDA at thresholds 0.4, 0.5, 0.6 detected 60%, 54% and 45% of seizures. At all thresholds, multivariate analyses demonstrated that the odds of detecting seizure increased with 4 criteria: seizure amplitude, duration, rhythmicity and number of EEG channels involved at seizure peak. Major causes of false detections included respiration and sweat artefacts or a highly rhythmic background, often during intermediate sleep.

**Conclusion:**

This rigorous analysis allows estimation of how key seizure features are exploited by SDAs.

**Significance:**

This study resulted in a beta version of ANSeR with significantly improved performance.

## Highlights

• A novel method for in-depth analysis of neonatal seizure detection algorithms is proposed.
• The analysis estimated how seizure features are exploited by automated detectors.
• This method led to significant improvement of the ANSeR algorithm.

## 1 Introduction

Full term neonates with neurological conditions such as hypoxic-ischaemic encephalopathy (HIE), stroke and meningitis are at high risk of developing seizures. There is accumulating evidence from animal models (Wirrell et al., 2001) and human studies (Glass et al., 2009) that neonatal seizures impose additional damage to the brain above and beyond the underlying aetiology. Prompt detection and treatment of seizures is therefore of paramount importance to optimize developmental outcome.

Clinical diagnosis of seizures is challenging, partly because clinically silent seizures can represent up to 85% of the total seizure burden (Bye and Flanagan, 1995) and over diagnosis based on clinical signs alone is common (Murray et al., 2008). Amplitude-integrated EEG (aEEG) is used in many neonatal intensive care units (NICUs), however comparison of seizure detection using EEG and aEEG has shown that many seizures seen on EEG are missed using aEEG alone (Rennie et al., 2004, Bourez-Swart et al., 2009).

It is now generally accepted that EEG is the only reliable means of accurately detecting all seizures in neonates and neonatal intensive care units are increasingly adopting prolonged EEG monitoring. These recordings may last hours or days, particularly for neonates with HIE who are cooled for 72 h. Although therapeutic hypothermia has been shown to reduce the seizure burden in this group (Low et al., 2012), seizures remain a problem. EEG is a complex signal that is prone to rhythmic artefacts which can mimic seizure patterns and requires highly trained experts to review and identify seizures. Experts are generally not available during unsociable hours and NICU staff generally lack EEG training and many feel unsupported in interpretation (Boylan et al., 2010). There is a high risk of both over and under diagnosis of seizures in neonates in the NICU.

There is therefore a pressing need to develop a reliable and robust automated seizure detection method for *full multi-channel* neonatal EEG. To meet this need, a novel automated seizure detection algorithm (SDA) has been developed for term neonates by our group (Mathieson et al., 2016), which is based on analyzing 55 features of seizures and using a support vector machine classifier for decision-making (Temko et al., 2011a). This algorithm (ANSeR) is currently undergoing clinical validation in NICUs across Europe in the ANSeR study (https://clinicaltrials.gov/ct2/show/NCT02160171).

In assessing the basic performance of SDAs engineers typically tend to produce ‘event’ based metrics including the percentage of seizures detected (seizure detection rate) and false detection rates, commonly quoted as false detections per hour (FD/hr). They may also use ‘epoch’ based metrics by segmenting the EEG to derive values for sensitivity (the amount of correctly identified seizure activity or seizure burden) and specificity (the amount of correctly identified non-seizure activity) (Temko et al., 2011b). While this primary analysis is essential, to fully understand the strengths and limitations of SDA performance and make informed modifications to improve performance, it is necessary to understand the characteristics or ‘nature’ of the seizures being missed, the specific causes of false detection and their relative contribution to the sum of false detections. Electrophysiologists, with an expert knowledge of neonatal EEG and the recording conditions in the NICU, are well placed to perform this type of analysis.

Although several algorithms have already been developed to automatically detect neonatal seizures, detailed analysis of this kind is often only anecdotally or partially discussed in previous performance assessment papers (Altenburg et al., 2003, Aarabi et al., 2006, Navakatikyan et al., 2006, Deburchgraeve et al., 2008, Mitra et al., 2009, Temko et al., 2011a, Mathieson et al., 2016).

The aim of this study was to introduce a comprehensive methodology for SDA performance analysis taking an electrophysiological approach by manually scoring *multiple* features of each seizure and examining differences in these features between detected and non-detected seizure groups and also fully characterizing and grouping all false detections and types of artefact when present. In this work the alpha version of the ANSeR algorithm was used as an example. The analysis of seizure features included initial univariate then multivariate analysis in order to assess whether a particular seizure feature was a determinant of seizure detection after the other features had been controlled for. This was done to identify areas for targeted improvement of the alpha version of the algorithm during the process of SDA development.

## 2 Methods

### 2.1 Automated seizure detection algorithm

**Fig. 1:**
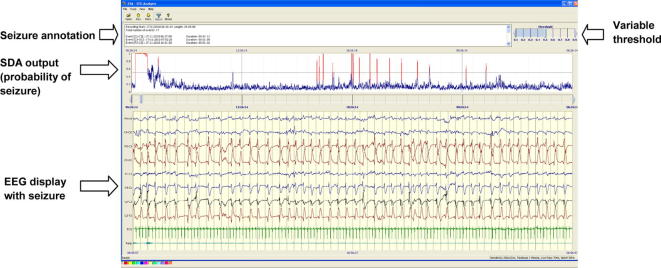
Automated seizure detection algorithm. Lower panel shows EEG reader displaying a seizure. Upper panel shows output of SDA. Blue trace is a graph of the probability of seizure. When the trace breeches an adjustable sensitivity threshold a seizure is designated, the trace turns red and an annotation of seizure time and duration is created.

A detailed description of the original alpha version of our SDA is given by Temko et al. (2011a). The EEG for each channel is initially pre-processed including filtering, artefact removal and segmentation into epochs. During the preprocessing step, simple high frequency artefacts are automatically removed by applying a threshold to the signal energy. Fifty-five features of the EEG are then extracted and the feature vectors fed into a support vector machine (SVM), a learning algorithm that has been pre-trained on EEG data containing seizures. The SVM outputs are converted using a sigmoid function into a probability of seizure between 0 and 1 for each epoch. The probability output is then smoothed by a moving average filter and compared to a threshold. Using the reader interface (Fig. 1) the threshold can be manually varied between 0 and 1 at intervals of 0.1. Comparison of the SVM probability output with the threshold is then converted into binary decisions, initially for each channel, then for all channels. The output has now been incorporated into a custom reader shown in Fig. 1. The EEG reader displays the EEG and the upper portion displays a graph showing the probability of seizure with the adjustable threshold, above which a seizure is classified (red) if breeched by the probability trace. The adjustable threshold allows the sensitivity of the algorithm to be manipulated to accommodate patients with high levels of artifact and false detection rates. The time, channel and duration of seizure are displayed and exportable as a text file.

### 2.2 EEG recording

EEG recordings on 20 term neonates, 10 with seizures and 10 seizure-free, were recorded in the neonatal units at University College Hospital, London and University College Hospital, Cork, Ireland. These 20 neonates were drawn as the first 20 cases from a randomized list of 70 neonates used in another study to validate standard performance metrics of the SDA (Mathieson et al., 2016), recorded between January 2009 and October 2011. Recordings were made using the NicoletOne monitor (Carefusion, Wisconsin, USA) using the 10:20 recording system adapted for neonates, using the following electrodes F4, F3, T4, T3, C4, C3, CZ, O2 and O1. The EEG was recorded at a sampling rate of 250/s or 256/s and with a filter bandwidth of 0.5–70 Hz and displayed using a bipolar montage.

### 2.3 Seizure analysis

All seizures were annotated on the original Nicolet EEG file at the beginning and end by an experienced electroencephalographer (SM) and the annotation list, which includes a quantification of seizure duration based on the annotations, was exported as a text file, which was then imported into Excel for further analysis. EEGs were identified by SM by reviewing the entire EEG page by page and seizures identified on the basis of electrographic evidence. Seizures annotated by SM were verified by a clinical neurophysiologist (RMP), blinded to patient details, by reviewing the EEG at the time of the annotation by SM. All recordings were also analyzed by the SDA (alpha version) post acquisition and text files of the SDA annotations for onset time, duration and channel of peak detection exported for three sensitivity thresholds (0.4, 0.5 and 0.6). From visual observation these were thought to include the most clinically relevant detection thresholds i.e. maximum number of detections with acceptable false detection rates.

The seizure annotations of SM were taken as the ‘gold standard’ for seizure detection. Seizure annotations were compared with those of the SDA to divide seizures into 2 groups, namely those *detected* and *non-detected* by the SDA. False detections were also separated for later classification.

Prior to determining which seizures were/were not detected by the SDA, all seizures detected by SM were individually quantified/scored under 10 criteria outlined in Table 1 for later comparison between the two groups of detected and non-detected seizures by the ANSeR SDA. In particular, seizure features analyzed included seizure signal signature features (1–5), temporal context or evolution of seizure (6–8) and seizure spatial context (9–10). Criteria for seizure morphology categorization and background pattern were adapted from Patrizi et al. (2003).

### 2.4 Statistical analysis

Univariate and multivariate mixed effects logistic regression analyses were performed to investigate and quantify the effects of seizure features on seizure detection. In the models, features were included as fixed effects and Baby ID as a random effect. The diagnostic accuracy of the models was assessed using the area under the receiver operator characteristic curve (AUC) and the corresponding 95% confidence interval (CI). Features that were statistically significant in the univariate analysis were candidate variables for the multivariate analysis. The features included in the final multivariate model were selected using backward stepwise deletion. Collinearity among the features was investigated prior to inclusion in the multivariate model and when collinearity was an issue, the feature with the highest AUC in the univariate analysis was included. Results are presented as odds ratios (OR) and 95% confidence intervals. The Mann–Whitney *U* test was used to compare the distribution of false detection rates between seizure and non-seizure neonates. Separate analyses were performed for each threshold. Statistical analysis was performed using Stata 13.0 (Texas, USA). All tests were two-sided and a *p*-value < 0.05 was considered to be statistically significant.

### 2.5 False detections

False detections (FD), defined as where the SDA had made a detection at the three SDA thresholds analysed that were not coincident with the seizure annotations of SM, were characterized and grouped under the following categories; respiration artefact, ECG/pulse artefact, chewing/sucking artefact, bad/loose electrode artefact, patient movement (including patting/stroking), electronic equipment artefact, sweat artefact, unclassified artefact, false detection with no obvious artefact. Where no artefact was detected during a false detection, a description of the EEG during the false detection was given under the headings; normal background; highly rhythmic EEG background, sharp waves, low amplitude EEG.

### 2.6 Ethical approval

Ethical approval was obtained for this study from the UCLH trust and the East London and the City Research Ethics Committee (REC reference number: 09/H0703/97) and by the Clinical Research Ethics Committees of the Cork Teaching Hospitals. Written informed consent was obtained from one parent of each neonate who participated in the study.

## 3 Results

### 3.1 Patients

Patient demographics are shown in Table 2.

### 3.2 Seizure detection and false detection rates

There were 421 seizures initially detected in a total of 1262.9 h of EEG (mean 63.1). RMP confirmed seizures in 419 of the 421 events annotated by SM (99.76%). The seizure detection/false detection rates for the SDA for seizure neonates are given in Supplementary Table S1a and the false detection rates for non-seizure neonates are given in Supplementary Table S1b. At lower thresholds (higher sensitivity) more seizures were detected but the false detection rate is also higher. Seizure detection rates and false detection rates fall as the sensitivity is decreased (threshold raised). False detection rates between seizure and non-seizure neonates were not significantly different at the 3 thresholds tested (threshold 0.4 *p* = 0.579, threshold 0.5 *p* = 0.280, threshold 0.6 *p* = 0.218).

### 3.3 Seizure features as predictors of automated seizure detection

The results of the univariate and multivariate analysis of seizure features as predictors of seizure detection for the three SDA sensitivity thresholds analysed are given in Table 3.

In the univariate analysis, for all 3 thresholds tested, 8/10 of the seizure features were a significant predictor of automated seizure detection. Higher peak amplitude, more frequency variability and rhythmicity and greater seizure duration and numbers of channels at seizure onset and seizure peak and change in morphology from start to peak of seizure were associated with increased odds of seizure detection. Seizure morphology at seizure peak was also a significant predictor of seizure detection. The odds of seizure detection was significantly higher in the spike and wave/sharp wave and slow wave complex (SP + W/SH + W) group compared to the rhythmic delta discharge (RDD) group at all thresholds. At threshold 0.6, the odds of seizure detection was also significantly higher in the sharp wave (SH) group compared to the rhythmic delta discharge (RDD) group. Seizure duration had the highest AUC across all thresholds (Supplementary Table S2).

Multivariate analysis was performed to determine if a particular feature remained a significant predictor of automated seizure detection after controlling for the other features. Collinearity was an issue between the seizure features “number of channels at seizure onset” and “number of channels at seizure peak” and hence only number of channels at seizure peak was included in the multivariate analysis, as this had the higher AUC in the univariate analysis. For all 3 thresholds tested, four of the features; seizure duration, amplitude, rhythmicity and number of EEG channels involved in the seizure at peak of seizure, were statistically significant predictors of seizure detection. Higher peak amplitude, more rhythmicity and greater seizure duration and numbers of channels at seizure peak were associated with increased odds of seizure detection. For thresholds 0.5 and 0.6, change in morphology from start to peak of seizure was also associated with increased odds of seizure detection. For threshold 0.4, higher frequency variability was associated with increased odds of seizure detection.

The AUCs (95% CI) for the multivariate model at all 3 ANSeR sensitivity thresholds was significantly better (threshold 0.4 *p* < 0.001, threshold 0.5 *p* < 0.001, threshold 0.6 *p* = 0.023) than the highest AUC in the corresponding univariate analysis (seizure duration) as shown in Supplementary Table S2, suggesting high accuracy of the multivariate model.

**Fig. 2:**
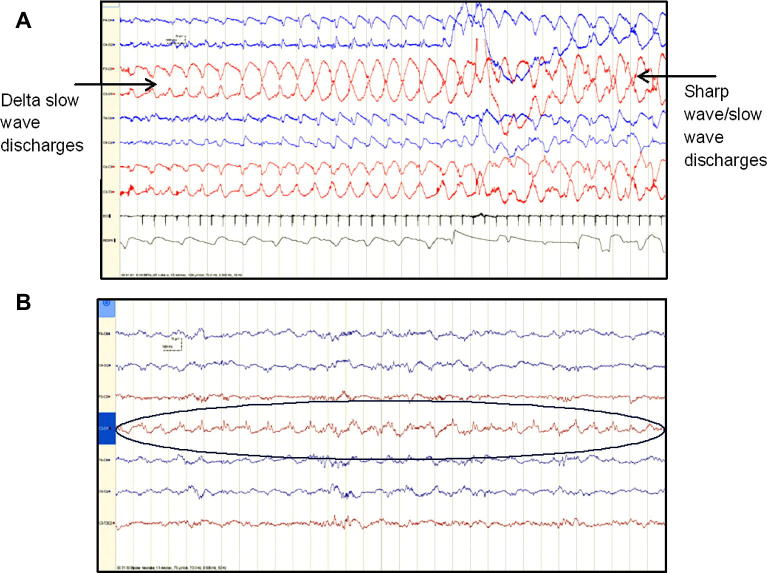
Typical detected/non-detected seizures. (A) Detected seizure- high amplitude, generalised, evolves from rhythmic delta discharges to sharp and slow wave complexes. (B) Non-detected seizure- low amplitude, no change in morphology or frequency, some dysrhythmia, single EEG channel.

Typical examples of detected seizures and non-detected seizures are shown in Fig. 2.

### 3.4 Categorization of false detections

The results of the categorization of false detections are shown in Table 4. For the 3 thresholds tested, respiration artefact was the most common cause of false detection followed by ‘no artefact identified’ and then sweat artefact. When false detections occurred and no artefact was identified, the background was often (approximately 59–65%) classified as highly rhythmic. Pulse/electrocardiogram artefact and movement/handling artefact also contributed to considerable numbers.

**Fig. 3:**
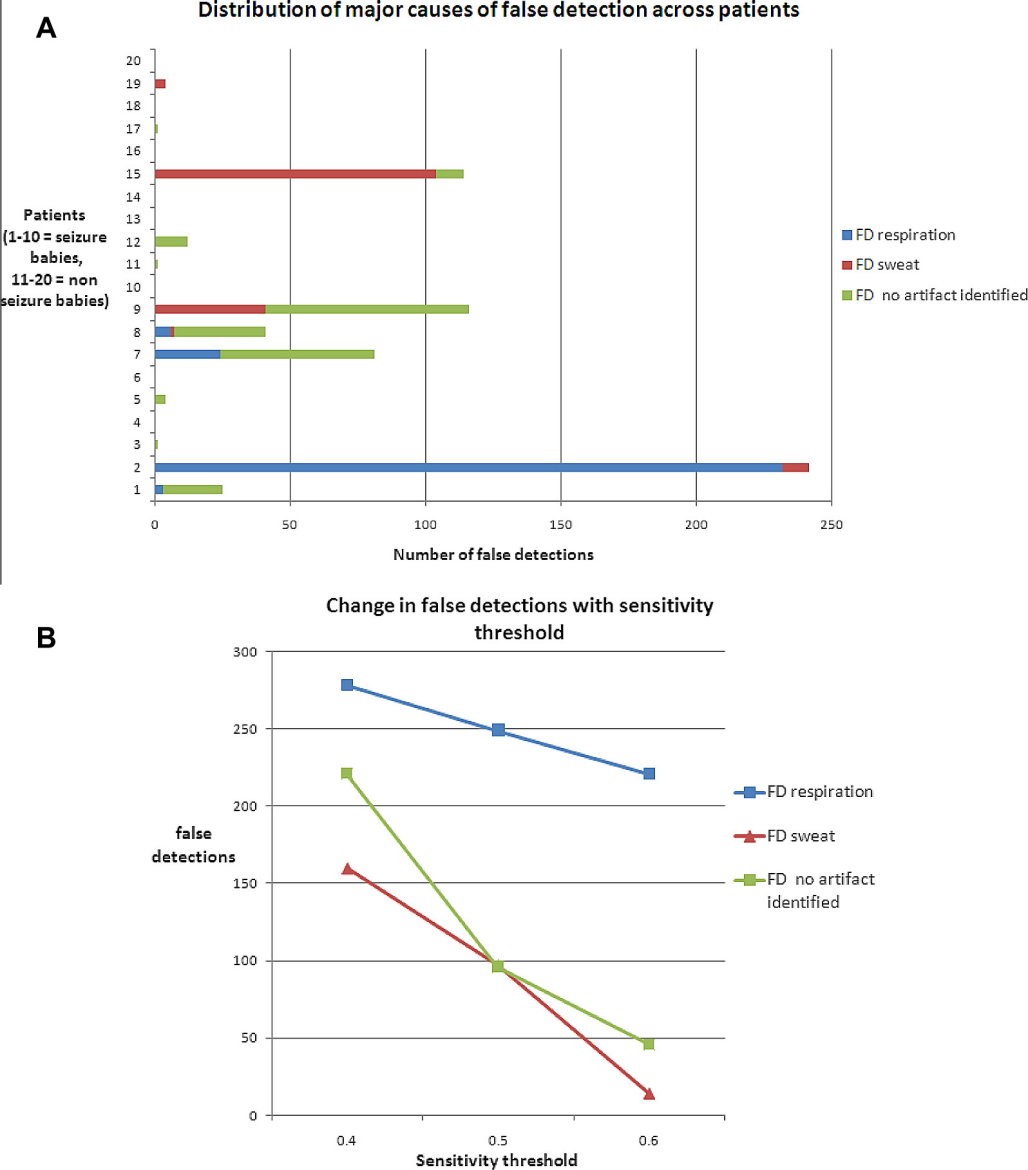
(A) Distribution of common causes of false detections. (B) Change in number of false detection with sensitivity threshold for the 3 main causes of false detection.

The distribution across patients of the three most prevalent causes of false detections for sensitivity threshold at 0.4 (Fig.3a) was not evenly spread and often a single patient recording was responsible for the majority of false detections in certain categories. For example 232/278 (83.5%) of false detections due to respiration artefact were seen in patient 2 and 104/149 (69.8%) of false detection due to sweat artefact were seen in patient 15. False detections where no artefact was identified (most often a highly rhythmic background EEG) were more distributed across several patients.

**Fig. 4:**
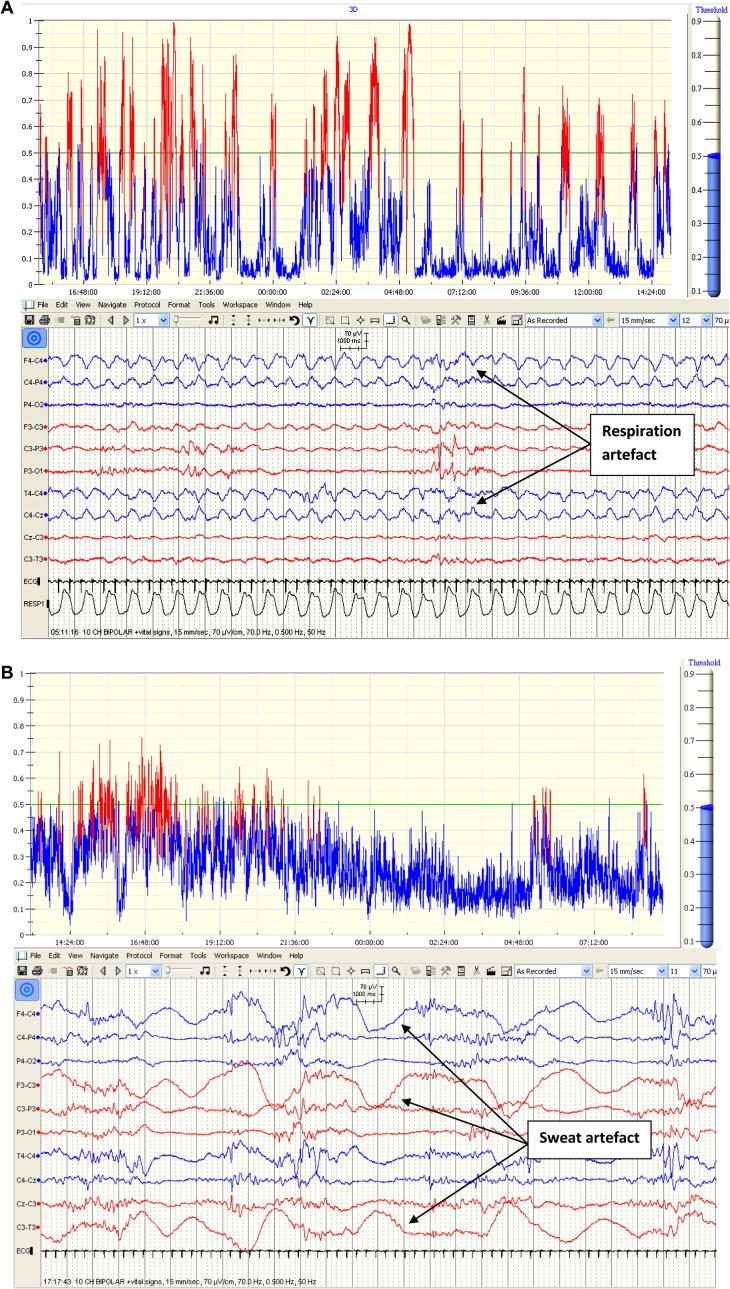
Effects of respiration and sweat artefacts on seizure probability output. (A) Highly rhythmic respiration artefact (lower panel) produces high probability peaks on SDA output graph (upper panel). (B) Intermittent semi-rhythmic slow sweat artefact on EEG (lower panel) produces a lower seizure probability output on graph (upper panel).

Fig.3b indicates how the number of false detections vary with SDA threshold sensitivity (0.4 = most sensitive, 0.6 = least sensitive) for the 3 most common causes. As expected, the number of false detections decreases as the sensitivity threshold increases (SDA becomes less sensitive). False detections due to respiration artefact show a moderate drop off with decreasing sensitivity while sweat and ‘no artefact detected’ false detection rates drop much more sharply with decreasing SDA sensitivity. The different rates of false detection drop-off are due to these waveforms generating different SDA seizure probability levels. For example, respiration artefact is a highly rhythmic artefact, closely mimicking seizure morphology, often resulting in high seizure probability output from the SDA (Fig.4a) whilst sweat artefact is a semi-rhythmic intermittent artefact generally producing a lower seizure probability (Fig.4b) thus as the sensitivity threshold is raised, a greater relative proportion of false detections remain under the threshold.

## 4 Discussion

This study sought to define a set of comprehensive criteria (Table 1) to analyse the characteristics of neonatal seizures to determine how the variability of these characteristics affected seizure detection of the SDA and to identify the key seizure features which were not exploited and main causes of false detections in order to identify areas for targeted improvement of the algorithms performance.

In previous SDA performance analysis studies, authors tend to only give anecdotal examples of missed seizures for their algorithms (Navakatikyan et al., 2006, Deburchgraeve et al., 2008), often described as short, arrhythmic, low amplitude or focal. Others have only examined the effect of a single parameter, seizure duration, on detection rate. Altenburg (Altenburg et al., 2003) found that their algorithm only detected seizures that were over 100 s in length. Assessments of the ANSeR algorithm (Temko et al., 2011a, Mathieson et al., 2016) similarly found that the poorest detection performance occurred when seizures were shortest (<1 min). Mitra (Mitra et al., 2009) gave more quantification describing missed seizures as either slow (>0.4 Hz) pseudosinoidal discharges (30%), high frequency (>6 Hz) with a depressed background (2%), arrhythmic spikes with a depressed background (15%) or short duration seizures (<20 s) in patients with longer seizures (53%).

Similarly, in terms of sources of false detection, authors only give subjective examples or descriptions. Aarabi (Aarabi et al., 2006) cited electromyogram, patient and electrode movement and signal saturations, while Deburchgraeve (Deburchgraeve et al., 2008) cited bed heater electrical artefact, ventilator or respiration artefacts or background rhythmicity as common sources of false detection. Others again gave some quantification of false detections; Mitra (Mitra et al., 2009) divided false detections into four groups; ‘rhythmic background’, ‘single channel’, ‘noisy data’ and ‘artefacts’. Temko et al. (2011a) similarly divided false detection into 3 groups; ‘artefact free background activity (50%), ‘artefacts’ (45%) and ‘seizure-like’ activity (5%) describing the most common forms of artefact causing false detection as ‘electrode disconnect’ (a slow semi-rhythmic high amplitude signal), ‘respiration artefact’ and ‘patient movement/handling artefact’. These authors however did not provide a quantitative breakdown of the relative contributions of *specific* artefacts to false detection rates. Some breakdown was given by Navakatikyan et al. (2006) stating that 39% of false detections were attributable to respiration *or* electrocardiogram/pulse artefact with rhythmic background theta activity causing a further 14% of false detections and ‘electrodes off’ artefact causing a further 15%.

Using the proposed methodology, the performance analysis of the ANSeR SDA presented in this study is in line with previous analysis (Temko et al., 2011a, Mathieson et al., 2016); as the SDA threshold is raised, seizure detection and false detection rates drop and there will always be a trade-off between picking a threshold that detects a satisfactory number of seizures whilst having an acceptable false detection rate. As the purpose of such an algorithm ultimately is to alert the clinical team to the presence of seizures, this trade off of which threshold is clinically acceptable can only really be tested in a clinical setting. However, this is the first study to provide an estimate of the contributions of key seizure features to detector’s behaviour.

The multivariate analysis in this study has shown that only four seizure features were consistent predictors of automated seizure detection across all three ANSeR sensitivity thresholds tested including: signal amplitude, the apparent rhythmicity of seizures from second to second, seizure duration, and the number of EEG channels involved in the seizure at the peak of seizure.

It is interesting to see that two of the four criteria come from the seizure signal signature group (Table 1). In fact, the ANSeR software relies on 55 features computed from the EEG signal that can be seen as universal EEG signal descriptors. Many of these features are energy-dependent and employ direct measures of amplitude such as root mean squared (RMS) amplitude and methods of spectral analysis during feature extraction such as total power and band power, where power is the square of the EEG amplitude. Thus seizure amplitude is expected to affect seizure detection rates.

Similarly, the increased rhythmicity of seizures from second to second as a predictor of seizure detection is in keeping with the findings of Mitra et al. (2009) and is expected as the SDA is tuned to detect distinct rhythms that stand out from the background. For example the ANSeR algorithm employs several measures of entropy at the feature extraction stage on the premise that background EEG with high complexity will have high entropy while seizures with a small number of dominant rhythms will have low entropy. Increased dysrhythmia in the seizure will increase the entropy and make it more similar to the background.

The fact that the longer seizure duration and the increased number of EEG channels involved at the seizure peak predicts increased automated detection has previously been reported (Altenburg et al., 2003, Navakatikyan et al., 2006, Deburchgraeve et al., 2008, Mitra et al., 2009). This observation is thus related to the computed metric rather than to the algorithmic solution. Indeed, a seizure is claimed to be detected if it is detected anywhere within the spatio-temporal manifold. For example, a 5 min long fully generalized seizure will be claimed detected if it is detected for only, say, 30 s in a single EEG channel. This clinically driven metric implies that increasing the number of involved channels and duration of seizure will statistically increase the chances of the seizure to be detected regardless of the content of the SDA.

Interestingly, most of the temporal context group from Table 1 were not found to be a predictor of the seizure consistently across different thresholds which clearly identifies the information that is currently not exploited in the detector. The key seizure features in this group such as increased frequency variability over the span of the seizures, change in seizure morphology (rhythmic delta to spike and wave/sharp wave and slow wave complexes) from start to peak of seizure characterize increased variability within a seizure *event.* On the contrary, the ANSeR SDA analyses 8 s overlapping EEG segments and changes of these two features within a given epoch are likely to be minimal as these changes tend to evolve gradually over time. Clearly, the short-term classification algorithm misses the information that is observable on a larger temporal scale in terms of the morphology change from rhythmic delta at the start of the seizure evolving to a spike and wave/sharp wave and slow wave morphology at the peak, and that is another potential area for improvement.

Fig.2a shows a detected seizure which is highly rhythmic, of high amplitude, involves multiple EEG channels and evolves in morphology from a rhythmic delta to sharp wave and slow wave complexes. In contrast, a typical non-detected seizure (shown in Fig.2b) is of shorter duration, lower amplitude, not changing in morphology, has a degree of dysrhythmia and only involving a single EEG channel.

Ultimately, these results suggest that the SDA should detect major seizures and may miss short, low amplitude seizures of arguably less clinical relevance.

If only a proportion of seizures are detected by the SDA and only a proportion are detected using aEEG, one might well ask what benefit there is of using the SDA instead of aEEG. Firstly in terms of seizures that are detected by the SDA, these will trigger and alarm and prompt clinicians to investigate the EEG at the time of the seizure or shortly after, leading to prompt administration of anticonvulsants. This is not true of the aEEG which, although provides a snapshot overview of the EEG, is still subject to the same periodic review as the EEG, such that seizure identification and treatment may be delayed. Secondly the aEEG will only ever register seizures that occur over the limited set of 2 or 4 electrodes from which it is generated. The SDA analyses a montage of 9 electrodes with a much broader coverage of the brain such that there is a greater potential to detect seizures. Even if seizures do not breech the SDA threshold and trigger an alarm, they may generate a peak on the SDA probability trend (which effectively summarizes all EEG channels as its output is the channel of highest probability) which can be used in the same way as the aEEG during periodic review to investigate areas of interest on the EEG. The aEEG is not only used for seizure detection and has additional important functions to provide a simple ‘snapshot’ assessment of brain function and the identification of sleep cycling. As such, the aEEG and SDA trend should both be viewed as valuable adjuncts to the EEG.

The analysis of false detections has highlighted several factors. Firstly it has highlighted the most common causes of false detection; namely respiration, sweat and a highly rhythmic background pattern. Respiration artefact was also cited by previous authors as a common source of false detection (Navakatikyan et al., 2006, Deburchgraeve et al., 2008, Temko et al., 2011a). A highly rhythmic background pattern was also a source of false detection for previous authors (Navakatikyan et al., 2006, Deburchgraeve et al., 2008, Mitra et al., 2009). Observationally, an increase in background rhythmicity was often associated with the ‘intermediate’ or ‘slow wave sleep’ pattern of quiet sleep in which an increase in semi-rhythmic background delta activity is observed.

Secondly, artefacts causing false detection may not be evenly spread across patients with some patients having high levels of artefact that will cause frequent false detection and alarms while other will have little or none. This suggests that the algorithm may perform less well for a small number of patients and better than expected for the majority. Implementing a variable sensitivity threshold in the SDA in the future should enable the user to desensitize the algorithm for patients with frequent false detections, although of course there will be a reduction in seizure detection performance. Thirdly, some artefacts such as respiration artefact are likely to be more persistent at higher sensitivity thresholds than others and along with prevalence, should be taken into account when prioritizing artefact rejection strategies.

Pulse/electrocardiogram artefact and movement/handling artefact also contributed significant numbers of false detections. Pulsatile artefact, caused by proximity of an electrode to a pulsing blood vessel, in particular, can cause very rhythmic runs of delta activity on the EEG mimicking seizures, and can be seen over prolonged periods. This artefact is identifiable as it will be fairly invariant and will be timelocked to the independently recorded ECG trace as will ECG artefact. Similarly, respiration artefact will be timelocked to the respiration trace recorded from the abdomen. Movement/handling artefacts, particularly those involving patting (winding), stroking or repetitive manipulations from physiotherapy, can create high amplitude and/or rhythmic artefacts on the EEG. Where these artefacts appear ‘seizure-like’ the video recording is invaluable in identifying these waveforms as artefact.

The reason for these artefacts or waveforms causing false detections is due to the fact that they constitute rhythmic stereotyped patterns, often with an increase in amplitude above the baseline, fulfilling many of the changes in frequency, power, amplitude, auto-regression, entropy and other parameters that the SDA is tuned to classify as seizures.

The proposed analysis of the ANSeR SDA performance outlined several areas of potential improvement. In particular, one key observation, that artefacts due to respiration, pulse and sweat often occur inprolonged runs, raising the probability baseline of the SDA output over a prolonged period, resulted in an adaptive modification which has been made to the alpha versions of the ANSeR SDA. The beta version now involves comparison of the probability graph at a given time to the ‘local’ preceding probability baseline. A comparison of the algorithm’s performance with/without the modification showed a significant increase in the area under the ROC curve from 93.4% to 96.1% and a reduced false detection rate from 0.42FD/hr (without adaption) to 0.24FD/hr (with adaption) while maintaining an equivalent detection of seizure burden at 70% (Temko et al., 2013). A further validation of the beta version of the SDA on a large set of 70 unedited EEGs has been published showing similar performance (Mathieson et al., 2016) which indicates the increased robustness of the ANSeR algorithm which was achieved as a byproduct of the analysis presented in this study.

Future studies will apply the same methodology outlined in this paper to the beta version of ANSeR, investigate the potential effect of anticonvulsants on seizures that persist and the performance of the SDA and aim to produce teaching material to improve the ability of users to discriminate true seizures from false detections at the point of SDA detection.

## 5 Conclusion

Due to the variability inherent in neonatal seizure and the numerous artefacts present in prolonged recordings in the intensive care environment, automated detection of neonatal seizure is a highly challenging problem. The analysis presented here has elucidated several aspects of the performance of the SDA from a neurophysiological perspective. In particular, it allows estimating the degree at which seizure relevant information is exploited in SDAs. The analysis applied to the ANSeR algorithm identified a number of directions for potential improvement and has since improved performance in the beta version of the ANSeR algorithm.

## Figures and Tables

**Table 1:** Seizure assessment criteria.

Variable group	Variable	Measurement type: quantitative/visual analysis	Measurement unit	Method/category	Purpose/comment
Seizure signature	Seizure amplitude at peak of seizure	Quantitative	μV^2^	Measure peak to trough using graticule on highest amplitude discharge at midpoint of seizure	To quantify the maximum seizure amplitude
Seizure signature	Rhythmicity score	Visual	Number	1 = significant dysrhythmia 2 = minimal dysrhythmia 3 = highly rhythmic	Visual score of how rhythmicity/frequency appears to change from second to second over the seizure
Seizure signature	Background EEG score at time of seizure	Visual	Number	1 = normal 2 = moderate abnormality 3 = severe abnormality ⁎see below	To highlight context in which seizure are detected/not detected
Seizure signature	Seizure morphology at onset	Visual	Category	1 = rhythmic discharges of delta (RDD) 2 = rhythmic discharges of theta (RDT) 3 = rhythmic discharges of alpha (RDA) 4 = spikes (S) or sharp waves (SH) 5 = sharp wave and slow wave (SH + W) complexes or spike and wave complexes (SP + W) ⁎see below	To categorize dominant morphology of seizure discharge at onset
Seizure signature	Seizure morphology at peak of seizure	Visual	Category	As above	To categorize dominant morphology of seizure discharge at peak (middle) of seizure
Short-term temporal context or evolution	Seizure duration	Quantitative	Seconds	Duration derived from SM annotations of start/end of seizure	To quantify seizure duration
Short-term temporal context or evolution	Frequency variability (over whole seizure)	Quantitative	SD (Hertz)	Using frequency graticule calculate discharge frequency at: A = start frequency (first 5 s) B = peak frequency (mid seizure) C = final frequency (last 5 s) Frequency variability = standard deviation A:C	To derive an estimate of the degree of frequency variability over the span of the seizure
Short-term temporal context or evolution	Seizure morphology change from onset to peak	Quantitative	Binary Y/N	Comparison of seizure morphology at start and peak	To assess change/variability of seizure morphology within seizure
Spatial context	Number of EEG channels involved at onset of seizure	Visual	Number	Count of number of EEG channels showing seizure discharges	To estimate the size of the seizure field at the start of the seizure
Spatial context	Number of EEG channels involved at peak of seizure	Visual	Number	Count of number of EEG channels showing seizure discharges	To estimate the size of the seizure field at the peak of the seizure

⁎ Adapted from Patrizi et al. (2003).

**Table 2:** Patients included in the study. HIE – hypoxic ischemic encephalopathy, MCA – middle cerebral artery, MAS – meconium aspiration syndrome, PPHN – persistent pulmonary hypotension, Pb – Phenobarbitone, Mdz inf – Midazolam infusion, Ptn – Phenytoin.

Patient	Electrographic seizures Y/N	Aetiology	Gestational age	Gender	Anti-epileptic medication	Morphine Y/N
1	Y	HIE grade 2	40 + 4	F	2 * Pb	N
2	Y	HIE grade 3	40 + 0	M	2 * Pb, Mdz inf	Y
3	Y	MAS, PPHN	40 + 5	F	Nil	Y
4	Y	Stroke	39 + 2	M	2 * Pb, Ptn	N
5	Y	Intraparenchymal haemorrhage	41 + 2	F	Pb	N
6	Y	Subdural haemorrhage	41 + 0	M	3 * Pb	N
7	Y	HIE grade 2	40 + 3	F	Pb	N
8	Y	Septic emboli ? Encephalitis	39 + 3	M	2 * Pb, Mdz inf	N
9	Y	Right MCA stroke	40 + 3	M	2 * Pb, Mdz inf	N
10	Y	Left haemorrhagic infarction	39 + 2	M	2 * Pb	N
11	N	HIE grade 1	39 + 3	F	Pb	N
12	N	Birth asphyxia	41 + 6	M	Nil	N
13	N	HIE grade 1	41 + 6	F	Nil	N
14	N	HIE grade 1	41 + 4	F	Nil	N
15	N	MAS	41 + 0	F	Nil	Y
16	N	HIE grade 1	41 + 2	M	Nil	N
17	N	HIE grade 2	41 + 4	M	Nil	N
18	N	HIE grade 2	39 + 1	M	Nil	Y
19	N	HIE grade 1	38 + 2	F	Nil	N
20	N	HIE grade 2	41 + 2	F	Pb	Y

**Table 3:** Univariate and multivariate mixed effects logistic regression analysis investigating seizure features associated with seizure detection.

	Threshold 0.4: logistic regression analysis						Threshold 0.5: logistic regression analysis						Threshold 0.6: logistic regression analysis					
Univariate analysis			Multivariate analysis			Univariate analysis			Multivariate analysis			Univariate analysis			Multivariate analysis		
Outcome: seizure detected	OR	(95% CI)	*p*-value	OR	(95% CI)	*p*-value	OR	(95% CI)	*p*-value	OR	(95% CI)	*p*-value	OR	(95% CI)	*p*-value	OR	(95% CI)	*p*-value
**Peak amplitude**	1.04	(1.03–1.05)	<0.001	1.02	(1.01–1.04)	<0.001	1.03	(1.03–1.04)	<0.001	1.02	(1.01–1.03)	<0.001	1.02	(1.01–1.02)	<0.001	1.01	(1.01–1.02)	<0.001
**Number of channels-seizure onset**	1.55	(1.28–1.88)	<0.001				1.6	(1.31–1.94)	<0.001				1.43	(1.19–1.71)	<0.001			
**Number of channels-seizure peak**	1.76	(1.46–2.13)	<0.001	1.46	(1.14–1.86)	0.002	1.79	(1.50–2.13)	<0.001	1.46	(1.15–1.86)	0.002	1.68	(1.43–1.98)	<0.001	1.35	(1.07–1.70)	0.011
**Rhymicity**			<0.001			0.004			<0.001			<0.001			<0.001			0.015
Significant dysrhythmia	1			1			1			1			1			1		
Minimal dysrhythmia	2.9	(1.57–5.38)		2.49	(1.08–5.75)		2.92	(1.58–5.38)		1.54	(0.69–3.45)		2.92	(1.49–5.72)		1.43	(0.58–3.56)	
Highly rhythmic	14.87	(7.07–31.25)		4.96	(1.93–12.78)		10.2	(5.21–19.98)		4.43	(1.91–10.24)		8.2	(4.13–16.26)		3.03	(1.21–7.60)	

**Seizure morphology-onset**			0.329						0.176						0.501			
RDD	1						1						1					
RDT	2.46	(0.68–8.84)					1.96	(0.78–4.93)					1.87	(0.78–4.48)				
RDA	3.64	(0.36–36.39)					3.8	(0.81–17.88)					2.74	(0.32–23.14)				
SH	0.67	(0.30–1.49)					0.81	(0.49–1.35)					0.8	(0.41–1.57)				
SH + W or SP + W	0.88	(0.49–1.58)					1.09	(0.68–1.74)					1.2	(0.70–2.03)				

**Seizure morphology-peak**			<0.001						<0.001						<0.001			
RDD	1						1						1					
RDT	7.46	(0.66–84.06)					1.21	(0.18–8.36)					1.33	(0.18–9.63)				
SH	1.85	(0.70–4.84)					2.51	(0.94–6.67)					3.33	(1.14–9.72)				
SH + W or SP + W	5.38	(2.45–11.78)					5.43	(2.46–12.02)					7.68	(3.20–18.46)				

**Change in morphology-start to peak**			<0.001						<0.001			0.012			<0.001			0.035
No	1						1			1			1			1		
Yes	3.23	(2.01–5.19)					2.75	(1.79–4.24)		2.33	(1.21–4.48)		2.31	(1.50–3.56)		2.02	(1.05–3.88)	
**Frequency variability**	19.46	(8.35–45.33)	<0.001	3.58	(1.32–9.66)	0.012	3.65	(2.05–6.48)	<0.001				2.54	(1.64–3.93)	<0.001			

**EEG background**			0.873						0.946						0.656			
Normal	1						1						1					
Mildly abnormal	0.85	(0.45–1.60)					0.93	(0.52–1.64)					1.32	(0.72–2.41)				
Severely abnormal	0.98	(0.25–3.82)					1.06	(0.39–2.91)					1.28	(0.41–3.98)				
**Seizure duration (secs)**	1.02	(1.02–1.03)	<0.001	1.02	(1.01–1.03)	<0.001	1.02	(1.02–1.03)	<0.001	1.02	(1.01–1.02)	<0.001	1.02	(1.02–1.03)	<0.001	1.02	(1.01–1.02)	<0.001

(1) Features with *p* > 0.05 in the univariate analysis were excluded from the multivariate analysis. (2) The multivariate model was selected using backward stepwise deletion. (3) The variable “Number of channels at seizure onset” was not included in the multivariate model due–colinearity with the feature “Number of channels at seizure peak”.

**Table 4:** Results of categorization of false detections. FD false detection. Numbers represent numbers of false detections for each category. Percentages in columns 2–8 represent percentage of overall false detections for each category. Where no artefact was identified on the EEG at the time of the false detection (column 8), a description of the background is given in column 9 (FD No artefact: comment). The percentages and numbers in column 9 therefore represent a breakdown of the totals in column 8.

SDA threshold	FD respiration artefact	FD ECG/Pulse artefact	FD bad electrode artefact	FD head movement /Handling artefact	FD sweat artefact	FD unclassified artefact	FD No artefact identified	FD No artefact: comment
0.4	278 (34.7%)	34 (4.2%)	21 (2.6%)	57 (7.1%)	160 (19.9%)	29 (3.6%)	221 (27.6%)	132 (59.73%) Highly rhythmic EEG, 67 (30.32%) normal background, 20 (9.05%) sharp waves, 2 (0.9%) low amplitude EEG
0.5	249 (47.9%)	42 (8.1%)	11 (2.1%)	16 (3.1%)	97 (18.7%)	19 (3.7%)	96 (18.5%)	55 (57.29%) Highly rhythmic EEG, 25 (26.04%) normal background, 15 (15.63%) sharp waves, 1 (1%) low amplitude EEG
0.6	221 (64.6%)	43 (12.5%)	4 (1.2%)	4 (1.2%)	14 (4.1%)	10 (2.9%)	46 (13.5%)	30 (65.22%) Highly rhythmic EEG, 11 (23.91%) normal background, 5 (10.87%) sharp waves
