# Supplementary material for: In-depth performance analysis of an EEG based neonatal seizure detection algorithm
Source: Clin Neurophysiol. 2016 May;127(5):2246–56. doi: 10.1016/j.clinph.2016.01.026 (PMC4840013; doi:10.1016/j.clinph.2016.01.026)
Supplement: Supplementary data 1 [file mmc1.docx]

| Patient number | Seizure detection rate thr 0.4 (%) | False detection rate the 0.4 (FD/HR) | Seizure detection rate thr 0.5 (%) | False detection rate thr 0.5 (FD/HR) | Seizure detection rate thr 0.6 (%) | False detection rate thr 0.6 (FD/HR) |
| --- | --- | --- | --- | --- | --- | --- |
| 1 | 79.17 | 0.39 | 66.66 | 0.06 | 54.17 | 0.01 |
| 2 | 52.63 | 1.73 | 52.63 | 1.53 | 47.37 | 1.43 |
| 3 | 28.57 | 0.03 | 28.57 | 0.02 | 28.57 | 0.00 |
| 4 | 95.83 | 0.00 | 83.33 | 0.00 | 79.17 | 0.00 |
| 5 | 77.14 | 0.65 | 74.29 | 0.31 | 60.00 | 0.18 |
| 6 | 20.00 | 0.00 | 0.00 | 0.00 | 0.00 | 0.00 |
| 7 | 60.00 | 0.93 | 50.00 | 0.48 | 30.00 | 0.24 |
| 8 | 60.71 | 0.88 | 57.14 | 0.64 | 57.14 | 0.53 |
| 9 | 74.10 | 1.11 | 56.12 | 0.60 | 44.44 | 0.28 |
| 10 | 58.54 | 0.02 | 52.85 | 0.00 | 38.21 | 0.00 |
| Median | 60.36 | 0.52 | 54.49 | 0.18 | 45.91 | 0.10 |

a

| Patient number | False detection rate thr 0.4 (FD/HR) | False detection rate thr 0.5 (FD/HR) | False detection rate thr 0.6 (FD/HR) |
| --- | --- | --- | --- |
| 11 | 0.06 | 0.00 | 0.00 |
| 12 | 0.60 | 0.20 | 0.04 |
| 13 | 0.02 | 0.00 | 0.00 |
| 14 | 0.08 | 0.00 | 0.00 |
| 15 | 5.63 | 3.75 | 0.64 |
| 16 | 0.00 | 0.00 | 0.00 |
| 17 | 0.01 | 0.00 | 0.00 |
| 18 | 0.05 | 0.05 | 0.00 |
| 19 | 0.65 | 0.14 | 0.00 |
| 20 | 0.08 | 0.04 | 0.03 |
| Median | 0.07 | 0.02 | 0.00 |

b
