# Supplementary material for: In-depth performance analysis of an EEG based neonatal seizure detection algorithm
Source: Clin Neurophysiol. 2016 May;127(5):2246–56. doi: 10.1016/j.clinph.2016.01.026 (PMC4840013; doi:10.1016/j.clinph.2016.01.026)
Supplement: Supplementary data 2 [file mmc2.docx]

| **AUC for logistic regression analysis** |  |  |  |  |  |  |  |  |
| --- | --- | --- | --- | --- | --- | --- | --- | --- |
|  | threshold | | | | | | | |
|  | 0.4 | |  | 0.5 | |  | 0.6 | |
| **Univariate analysis** | AUC | (95% CI) |  | AUC | (95% CI) |  | AUC | (95% CI) |
| Peak amplitude | 0.84 | (0.80 to 0.88) |  | 0.82 | (0.78 to 0.86) |  | 0.79 | (0.75 to 0.83) |
| Number of channels at seizure onset | 0.71 | (0.67 to 0.76) |  | 0.68 | (0.63 to 0.72) |  | 0.67 | (0.62 to 0.71) |
| Number of channels at seizure peak | 0.73 | (0.69 to 0.77) |  | 0.68 | (0.63 to 0.72) |  | 0.68 | (0.63 to 0.72) |
| Rhymicity | 0.78 | (0.74 to 0.82) |  | 0.74 | (0.69 to 0.78) |  | 0.72 | (0.68 to 0.77) |
| Seizure morphology at seizure onset | 0.66 | (0.62 to 0.71) |  | 0.56 | (0.51 to 0.60) |  | 0.61 | (0.57 to 0.66) |
| Seizure morphology at seizure peak | 0.72 | (0.68 to 0.76) |  | 0.68 | (0.63 to 0.72) |  | 0.70 | (0.65 to 0.74) |
| Change in morphology from start to peak | 0.71 | (0.66 to 0.75) |  | 0.66 | (0.62 to 0.71) |  | 0.66 | (0.61 to 0.70) |
| Frequency variability | 0.79 | (0.75 to 0.83) |  | 0.73 | (0.68 to 0.77) |  | 0.72 | (0.67 to 0.76) |
| EEG background | 0.66 | (0.61 to 0.70) |  | 0.61 | (0.56 to 0.65) |  | 0.63 | (0.58 to 0.68) |
| Seizure duration (secs) | 0.88 | (0.85 to 0.91) |  | 0.87 | (0.83 to 0.90) |  | 0.89 | (0.85 to 0.92) |
| **Multivariate analysis** | 0.93 | (0.90 to 0.95) |  | 0.92 | (0.89 to 0.94) |  | 0.91 | (0.88 to 0.94) |
